# Factors associated with hepatitis C prevalence differ by the stage of liver fibrosis: A cross-sectional study in the general population in Poland, 2012-2016

**DOI:** 10.1371/journal.pone.0185055

**Published:** 2017-09-20

**Authors:** Magdalena Rosińska, Natalia Parda, Agnieszka Kołakowska, Paulina Godzik, Karolina Zakrzewska, Kazimierz Madaliński, Andrzej Zieliński, Anna Boguradzka, Rafał Gierczyński, Małgorzata Stępień

**Affiliations:** 1 Department of Epidemiology, National Institute of Public Health-National Institute of Hygiene, Warsaw, Poland; 2 Department of Virology, National Institute of Public Health-National Institute of Hygiene, Warsaw, Poland; 3 Department of Family Practice, Centre of Postgraduate Medical Education, Warsaw, Poland; 4 Department of Bacteriology, National Institute of Public Health-National Institute of Hygiene, Warsaw, Poland; Centers for Disease Control and Prevention, UNITED STATES

## Abstract

**Background & aims:**

There is a considerable burden of hepatitis C in Europe related to the lack of prompt diagnosis. We aimed to estimate the prevalence and related risk factors of HCV infections by the stages of liver fibrosis, using non-invasive methods, to understand testing needs in Poland.

**Methods:**

A cross-sectional study was conducted in 2012–2016 adopting a stratified random sampling of primary health care units followed by systematic sampling of patients within each unit. Study participants filled a questionnaire and donated blood for laboratory HCV testing. Additionally, the results of liver function tests and platelet count were collected to calculate APRI and FIB-4 scores. Cases were classified according to the level of fibrosis: ‘significant fibrosis’ (APRI≥0.7 or FIB4≥1.45) and ‘no significant fibrosis’ (APRI<0.7 and FIB4<1.45).

**Results:**

Of 21 875 study participants, 102 were HCV-RNA positive. Prevalence of HCV infections and significant fibrosis was estimated at 0.47% (95% CI 0.38% - 0.57%) and 0.12% (0.08% - 0.17%), respectively. Cases with significant fibrosis accounted for 51.6% (33.4%-69.9%) in men and 34.4% (17.3%-51.4%) in women. There was no correlation between the HCV prevalence and age. Blood transfusion prior to 1992 strongly predicted significant fibrosis as did the history of injecting drug use (IDU) and ever having an HCV-infected sexual partner in men and caesarean sections in women. Factors associated with HCV infection without significant fibrosis were tattooing in men and younger age in women. We acknowledge limited possibility to study the associations between IDU and ever having HCV-infected sexual partner, given small sample sizes for these exposures.

**Conclusions:**

As no clear birth cohort affected by HCV could be identified, risk factor-based screening in the general population should be considered, taking into account the association between the increased risk of liver fibrosis and the history of transfusion prior to 1992 and caesarean sections.

## Introduction

Hepatitis C is considered to be one of the most important public health problems related to the infectious diseases worldwide. Severe complications and mortality of this disease generate high economic and societal costs [1,2].

Simultaneously, there is a unique opportunity to avoid the unfavourable outcomes of this condition, including liver cirrhosis and hepatocellular carcinoma, with the advent of new potent therapies [3]. Current therapeutic regimens allow for a successful treatment of hepatitis C cases even with advanced liver fibrosis which previously constituted a contraindication to interferon-based therapies [4,5]. Furthermore, although treatment is currently recommended for all patients with chronic HCV infection, patients with significant liver fibrosis are prioritized according to the European guidelines [6].

In all cases, the success of therapeutic programmes in curbing the epidemic heavily relies on the effectiveness of case detection. Recently, a substantial effort was undertaken to understand the epidemiology of hepatitis C in order to design and implement appropriate screening programmes [7,8]. Different scenarios were evaluated for cost–effectiveness ranging from targeted screening of high risk groups (e.g. people who inject drugs, PWID) to population-based screening of birth cohorts [9]. The example of the USA shows that screening of appropriate birth cohorts in addition to risk factor-based screening may be effective in detecting the majority of chronic infections without a substantial increase of the costs [10]. Of importance, a relatively small birth cohort could be identified which comprises over 75% of chronic HCV infections in the USA, i.e. people born in 1945–1965 (‘baby boomers’).

Substantial prevalence of hepatitis C was also confirmed in several studies conducted in Poland [11–14]. However, risk factors for viraemic prevalence were not established. Moreover, the distribution of HCV-related fibrosis in prevalent cases is not known. This could affect short-term predictions of the burden of this disease.

The objective of our study was to estimate the prevalence of HCV infections and the proportion of people with HCV-related fibrosis, using non-invasive methods. Furthermore, we aimed at identifying risk factors associated with higher HCV prevalence and more advanced fibrosis stage as to inform public health authorities of the groups in which rapid improvement of the detection rate is needed.

## Materials and methods

### Study design

We conducted a cross-sectional study in the general population in Poland. The sampling frame was defined as the population registered in the primary health care units. At the time of the study, the coverage of primary health care was estimated at about 84% (Annual Report of the National Health Fund 2014, http://www.nfz.gov.pl/zarzadzenia-prezesa/uchwaly-rady-nfz/). Participants were selected using multi-stage sampling strategy. Firstly, a random sample of primary health care units stratified by region was drawn from the primary health care unit database. This database was derived from the Register of Health Care Centres maintained by the Centre for Health Information Systems, containing both public and non-public units. Units from 10 out of 16 provinces were sampled and 45 units (3 to 6 per province) agreed to participate in the study. In each unit, a systematic sample was drawn from the register of patients, according to the number of potential participants declared by the unit personnel. Patients were contacted by phone, post or face-to-face. Detailed information of the sampling and recruitment procedure was specified in [15].

Inclusion criteria for patients were: at least 18 years of age, not being currently on treatment for hepatitis C and signed written informed consent. One of the contacted individuals was excluded because of being treated for HCV infection. The overall response rate was 85%.

Each study participant was asked to fill an anonymous questionnaire and donate blood for laboratory testing for HCV. The questionnaire was self-completed. The participants, however, were encouraged to discuss particular questions with the general practitioner during the visit and update answers if appropriate. Laboratory results and questionnaires were processed separately and linked by an ID code in the central database.

In case of positive HCV-RNA result, the patient was asked to attend a follow-up visit to undergo additional tests and receive referral to a specialist. Additional information was collected at this stage including the results of liver function tests and platelet count.

The process of data collection lasted from March 2013 to April 2016. The protocol was approved by the Ethics Committee at the National Institute of Public Health–National Institute of Hygiene in Warsaw (NIPH-NIH).

### Questionnaire

The questionnaire was developed at the Department of Epidemiology of the NIPH-NIH. It was subject to a pilot study in a selected primary health care unit in Warsaw. The questionnaire included the questions on demographics, general medical history (e.g. comorbidities, prior testing for HCV), medical and non-medical exposures (e.g. drug use, beauty procedures including tattoos, home or sexual contact with a known HCV case, history of arrest, history of alcohol addiction treatment). The original questionnaire in Polish and the English translation are available as S1 File and S2 File, respectively.

### Laboratory methods

Blood samples collected from the participants were processed at the local laboratories according to a uniform algorithm (centrifugation at least 2000 RPM for 10 minutes, aliquoting into 2 tubes and freezing at max. -20°C). Frozen blood samples were transported to the Department of Virology of the NIPH-NIH at two-week intervals at most.

All serum samples were initially tested using electro-chemiluminescence immunoassay (ECLIA) (Anti-HCV II, Cobas e 411, Roche). Samples that were repeatedly reactive were subject to polymerase chain reaction (PCR) for HCV-RNA (artus®RG RT-PCR Kit, Qiagen). Testing was performed according to the manufacturer’s instructions.

### Statistical analysis

#### Definition of the outcome variable

The patients were classified as infected according to the HCV-RNA result. ‘Significant fibrosis’ was assigned to individuals who had high scores in APRI (aminotransferase–platelet ration index) or FIB4 indices. The individuals with low scores were classified as ‘no significant fibrosis’.

APRI is calculated using the following formula: APRI = (AST [U/L]/Upper limit of normal AST [U/L])/ PLT[10^9^/L].

FIB4 is calculated as follows: FIB4 = (age[years]xAST[U/L])/(PLT[10^9^/L]x (ALT[U/L])^0.5^).

Based on the available literature, we used the following cut-offs for significant fibrosis: APRI ≥0.7 [16,17] and FIB4≥1.45 [18]. In a recent meta-analysis, APRI score of above 0.7 had a sensitivity of 77% and the specificity of 72% to detect significant fibrosis defined as METAVIR, Batts and Ludwig, or Scheuer stages F2 through F4 or Ishak and colleagues stages F3 through F6 [17]. Using the 1.45 cut-off for FIB4 allowed this test to have a sensitivity of 70% and specificity of 73.7% to detect significant fibrosis (Ishak F3 –F6)[18].

#### Approach to missing data

The proportion of missing data in the questionnaires was relatively low. However, the results of platelet count and liver function tests were collected during follow-up visit and, consequently, they were not available for all patients. Having assumed that missing information is not related to the actual value of the parameter (missing at random), we applied multiple imputations of the values taking into account the exposures considered in the substantial model as well as chronic conditions described in the literature to be associated with either platelet count or AST or ALT levels. Fully conditional specification (chained equations) technique [19] was used to create the imputations using normal regression model to the platelet count and natural logarithm transform of AST and ALT levels. Other parameters were modelled using logistic regression. Ten pseudo-complete datasets were created and the Rubin’s rules were used for combined inference [20].

#### Multivariate analysis of factors associated with HCV infection

We used logistic regression to study the association between demographic factors, medical and non-medical exposures and HCV infection and multinomial regression model to study the association between these factors and ‘no significant fibrosis’ and ‘significant fibrosis’ versus the baseline category ‘no active infection’. Due to the differential risk of HCV elimination and prognosis of disease progression in men and women, the model was developed separately for men and women. Initially, the effects significant in univariable analysis were considered. Backward selection was applied to achieve the final model. The analysis was performed using STATA (StataCorp. 2013. Stata: Release 13. Statistical Software. College Station, TX: StataCorp LP).

## Results

**Fig 1:**
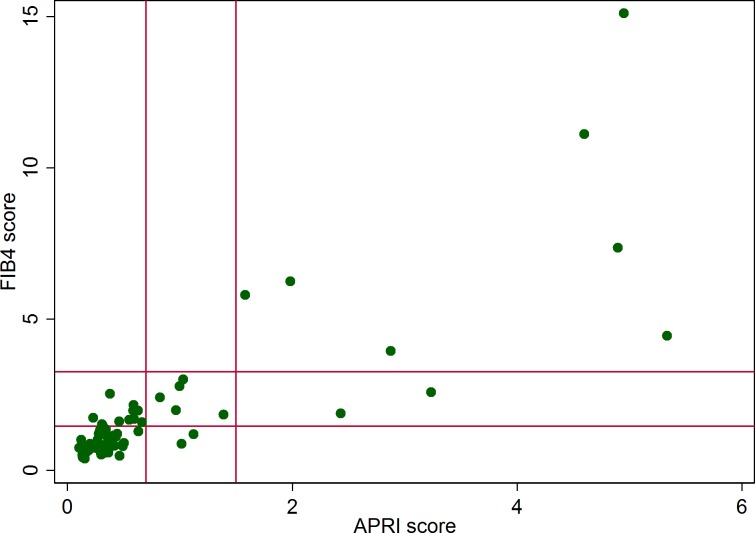
Comparison of APRI and FIB4 scores in HCV-RNA positive individuals. The vertical and horizontal lines indicate the cut-off points for significant fibrosis for APRI and FIB4 scores, respectively. Two cut-off points are presented: APRI≥0.7 and FIB4≥1.45 (high sensitivity and low specificity) and APRI≥1.5 and FIB4≥3.25 (low sensitivity and high specificity).

In total, 21 875 individuals were included in the study. Presence of HCV-RNA was confirmed in 102 cases. The information necessary to calculate ARPI and FIB4 scores was available for 63 cases. A total of 36 (57.1%) and 14 (22.2%) cases were classified both by APRI and FIB4 scores as having low and high probability of significant fibrosis, respectively (Fig 1). These two indices were discrepant in 13 cases (20.6%), that, according to our procedure, were assigned to the significant fibrosis group.

A total of 14 295 (65.3%) women and 7 580 (34.7%) men were included in the study. The median age of the participants was 49.5 years (IQR 36–60), 50.2 years (IQR 36.4–60.5) in men and 49.1 years (IQR 35.8–59.7) in women. The distributions of socio-demographic factors, medical and non-medical factors in all participants, the infected individuals and those with the indication of significant fibrosis are given in Table 1. Of note, blood transfusion prior to 1992 was recorded in 5.4% of all participants, 16.7% of infected individuals and 25.9% of those with significant fibrosis. Similarly, 0.3% of the participants, 4.9% of infected individuals and 3.7% of those with significant fibrosis reported history of injecting drug use. A total of 0.4%, 3.9% and 3.7% marked contacts with an HCV-infected sexual partner, respectively. Tattooing was reported by 5.7% of participants and 12.7% of infected individuals. Notably, tattooing was reported by 10.2% of people aged 18–44 (873 of 8,605 individuals who provided a valid answer) and 3.2% (374/11,789) of those aged 45 or older. Out of all women, 17.4% confirmed prior caesarean section as compared to 35.3% of HCV-infected women.

**Table 1:** Characteristics of the study population, HCV-infected individuals and individuals with indication of significant liver fibrosis.

	Total		HCV-RNA positive		Significant fibrosis	
	No.	Col %	No.	Col %	No.	Col %
**Sex**						
**Women**	14295	65.3	51	50.0	11	40.7
**Men**	7580	34.7	51	50.0	16	59.3
**Age mean/median, IQR (years)**	48.5/ 49.5,30–60		47/ 47.6, 33.2–59.1		53.7 / 52.4, 44–69.0	
**Residence**						
**Rural**	10353	47.3	39	38.2	12	44.4
**Urban**	11522	52.7	63	61.8	15	55.6
**Transfusion prior to 1992**						
**No**	20231	92.5	85	83.3	20	74.1
**Yes**	1171	5.4	17	16.7	7	25.9
**N.A.**	473	2.2	0	0.0	0	0.0
**Dental procedure**						
**No**	3184	14.6	12	11.8	5	18.5
**Yes**	16998	77.7	81	79.4	19	70.4
**N.A.**	1693	7.7	9	8.8	3	11.1
**Biopsy**						
**No**	15442	70.6	70	68.6	21	77.8
**Yes**	3166	14.5	21	20.6	4	14.8
**N.A.**	3267	14.9	11	10.8	2	7.4
**Caesarean section**						
**No**	9574	67.0	29	56.9	6	54.5
**Yes**	2486	17.4	18	35.3	4	36.4
**N.A.**	2235	15.6	4	7.8	1	9.1
**Total**	14295	100.0	51	100.0	11	100.0
**Major surgery**						
**No**	10628	48.6	52	51.0	13	48.1
**Yes**	8511	38.9	39	38.2	12	44.4
**N.A.**	2736	12.5	11	10.8	2	7.4
**Tattoos**						
**No**	19147	87.5	86	84.3	25	92.6
**Yes**	1247	5.7	13	12.7	2	7.4
**N.A.**	1481	6.8	3	2.9	0	0.0
**Treatment for alcohol addiction**						
**No**	21282	97.3	95	93.1	26	96.3
**Yes**	265	1.2	4	3.9	1	3.7
**N.A.**	328	1.5	3	2.9	0	0.0
**Intranasal or injecting drug use**						
**No**	21000	96.0	93	91.2	26	96.3
**Only intranasal**	180	0.8	2	2.0	0	0
**Injecting**	61	0.3	5	4.9	1	3.7
**N.A.**	634	2.9	2	2.0	0	0.0
**HCV diagnosed in householder**						
**No**	20874	95.4	98	96.1	26	96.3
**Yes**	615	2.8	4	3.9	1	3.7
**N.A.**	386	1.8	0	0.0	0	0.0
**HCV-infected sexual partner**						
**No**	21482	98.2	98	96.1	26	96.3
**Yes**	94	0.4	4	3.9	1	3.7
**N.A.**	299	1.4	0	0.0	0	0.0
**Total**	21875	100.0	102	100.0	27	100.0

Having imputed missing data, the overall prevalence of HCV-RNA was 0.47% (95% CI 0.38% - 0.57%) and the prevalence of significant fibrosis was 0.12% (0.08% - 0.17%). The cases with significant fibrosis accounted for 42.9% (30.3%-55.4%) of all infections, 51.6% (33.4%-69.9%) in men and 34.4% (17.3%-51.4%) in women.

**Fig 2:**
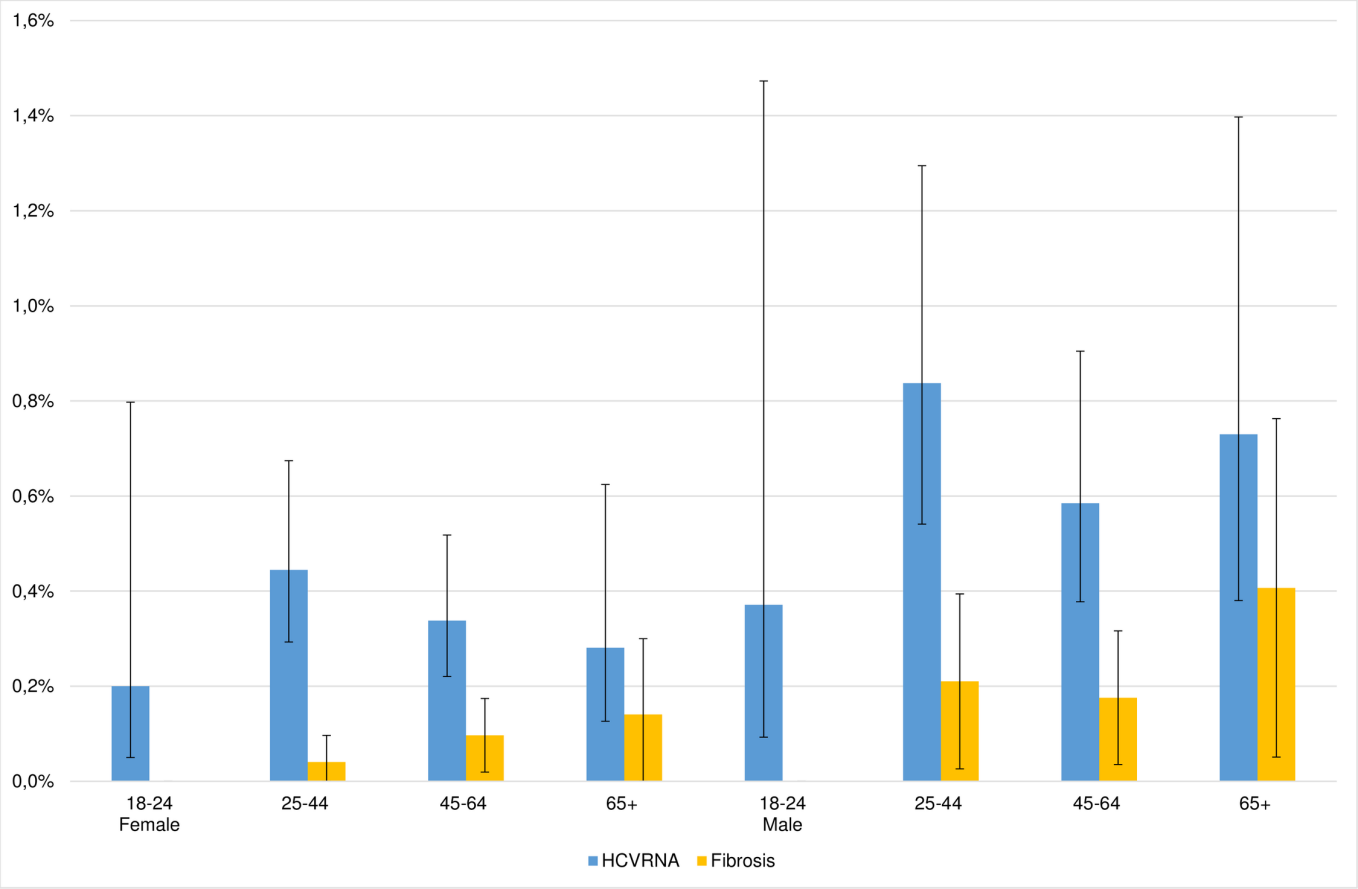
Prevalence of HCV-RNA and prevalence of significant fibrosis defined by APRI≥0.7 and FIB4≥1.45 by sex and age group.

Both the prevalence of active infections and significant fibrosis were higher in men compared to women (0.67%, 95% CI 0.51% - 0.88% vs 0.36%, 95% CI 0.27%-0.47% and 0.21%, 95% CI 0.11%-0.32% vs 0.08%, 95% CI 0.03%-0.12%, respectively). The prevalence of significant fibrosis increased with age, but the differences in HCV prevalence were not significant (Fig 2).

Initially, we studied all factors in the multivariable regression models, which in univariable analysis predicted either HCV infection in general, ‘no significant fibrosis’ infection or ‘significant fibrosis’ infection either in men or women at the level of 0.1. Due to the low number of cases (or no cases) in some categories, respective odds ratios were not possible to calculate. In the multivariable model, we retained factors with p-values<0.1.

In both men and women, HCV infection was associated with blood transfusion prior to 1992 (adjusted odds ratio, AOR 4.6, 95% CI 1.83–11.59 and 3.5, 95%CI 1.7–7.21, respectively) and ever having an HCV-infected sexual partner (AOR 7.26, 95% CI 1.59–33.25 and 9.88, 2.3–42.51) (Table 2). In addition, caesarean sections predicted HCV infection in women (AOR for ≥2 caesarean sections vs none 3.4, 95% CI 1.68–6.89) and injecting drug use (AOR 10.09, 95% CI 3.08–33.04), having a tattoo (AOR 2.58, 95% CI 1.28–5.2) and a history of biopsy (AOR 3.72, 95% CI 1.93–7.19) in men.

**Table 2:** Univariate and multivariate associations between predictive factors and HCV infection by sex.

	Univariable				Multivariable*			
	OR	95% CI		p-value	AOR	95% CI		p-value
**WOMEN**								
**Age (per each additional year)**	0.99	0.98	1.01	0.479	0.99	0.97	1.01	0.216
**Transfusion prior to 1992 (yes vs no)**	3.58	1.83	6.99	<0.001	3.5	1.7	7.21	0.001
**Having a tattoo (yes vs no)**	0.78	0.19	3.23	0.735				
**Ever using intranasal or injecting drugs (yes vs no)**	7.21	1.72	30.19	0.007				
**Ever injecting drugs (yes vs no)**	9.91	1.32	74.25	0.026				
**Biopsy (yes vs no)**	0.72	0.31	1.67	0.448				
**HCV positive partner (yes vs no)**	9.8	2.33	41.25	0.002	9.88	2.3	42.51	0.002
**Dental procedure (yes vs no)**	1.09	0.5	2.41	0.825				
**Perinatal procedures (yes vs no)**	0.79	0.45	1.4	0.426				
**Alcohol addiction (yes vs no)**	4.61	0.63	33.93	0.133				
**HCV positive household contact (yes vs no)**	1.25	0.3	5.17	0.754				
**One caesarean section vs none**	1.46	0.66	3.24	0.353	1.24	0.55	2.78	0.609
**Two or more caesarean sections vs none**	4.03	2.02	8.03	<0.001	3.4	1.68	6.89	0.001
**MEN**								
**Age (per each additional year)**	0.99	0.98	1.01	0.459	0.99	0.97	1.01	0.172
**Transfusion prior to 1992 (yes vs no)**	5.52	2.32	13.12	<0.001	4.6	1.83	11.59	0.001
**Having a tattoo (yes vs no)**	3.19	1.63	6.26	0.001	2.58	1.28	5.2	0.008
**Ever using intranasal or injecting drugs (yes vs no)**	5.1	2	13.01	0.001				
**Ever injecting drugs (yes vs no)**	21.87	7.38	64.84	<0.001	10.09	3.08	33.04	<0.0001
**Biopsy (yes vs no)**	3.74	2.01	6.99	<0.001	3.72	1.93	7.19	<0.0001
**HCV positive partner (yes vs no)**	8.92	2.08	38.17	0.003	7.26	1.59	33.25	0.011
**Dental procedure (yes vs no)**	1.39	0.56	3.49	0.479				
**Alcohol addiction (yes vs no)**	2.23	0.69	7.23	0.18				
**Ever having an HCV positive household contact (yes vs no)**	1.73	0.42	7.19	0.448				

*Combined estimates of logistic regression on 10 imputed datasets.

The only risk factor significantly associated with significant fibrosis in women was the number of caesarean sections, AOR for two or more vs none 5.6 (95% CI 1.3–24). Transfusion prior to 1992 was only borderline significant in women in multivariable analysis, although strongly predicted significant fibrosis in univariable analysis (OR 4.9, 95% CI 1.3–18.5) (Table 3).

**Table 3:** Univariate and multivariate associations between predictive factors and HCV infection with ‘significant fibrosis’ (defined by APRI≥0.7 and/or FIB4≥1.45) and ‘no significant fibrosis’ by sex.

	Univariable								Multivariable**							
	OR no significant fibrosis*	95% CI		p-value	OR significant fibrosis	95% CI		p-value	AOR no significant fibrosis*	95% CI		p-value	AOR significant fibrosis	95% CI		p-value
**WOMEN**																
**Age (per year)**	0.97	0.94	1	0.042	1.03	0.99	1.07	0.101	0.96	0.93	0.99	0.021	1.03	0.99	1.08	0.129
**Transfusion prior to 1992 (yes vs no)**	2.2	0.6	7.4	0.213	4.9	1.3	18.5	0.019	3.3	0.9	12.2	0.074	2.9	0.7	11.9	0.129
**Biopsy (yes vs no)**	0.4	0.1	1.8	0.224	0.5	0.1	3.7	0.482								
**One caesarean section vs none**	1.2	0.3	4	0.82	1.3	0.2	10.2	0.807	1	0.3	3.6	0.957	1.3	0.2	10.7	0.796
**Two or more caesarean sections vs none**	1.4	0.3	6.3	0.631	5.7	1.4	23.2	0.014	1.3	0.3	5.6	0.758	5.6	1.3	24	0.021
**MEN**																
**Age (per year)**	0.97	0.94	1.01	0.109	1.01	0.98	1.05	0.417	0.97	0.94	1.01	0.141	1.01	0.97	1.04	0.665
**Transfusion prior to 1992 (yes vs no)**	3.0	0.4	22.6	0.296	13.8	4.4	43.2	<0.0001	3.4	0.4	27.8	0.246	12.1	3.7	39.9	<0.0001
**Having a tattoo (yes vs no)**	5.8	2.0	17.0	0.0001	1.7	0.4	7.3	0.504	4.6	1.6	13.9	0.006	1.4	0.3	6.6	0.645
**Ever using intranasal or injecting drugs (yes vs no)**	7.2	1.6	32.3	0.01	3.1	0.4	23.8	0.278								
**Ever injecting drugs (yes vs no)**	18.4	2.3	144.4	0.006	17.1	2.2	134.2	0.007	8.6	1.0	74	0.049	8.2	0.9	73.8	0.059
**Biopsy (yes vs no)**	2.4	0.7	8.5	0.18	2.5	0.7	9.1	0.15								
**HCV positive partner (yes vs no)**	15.6	2.0	122.1	0.009	14.6	1.9	113.4	0.011	9.6	1.2	79.3	0.036	11.6	1.4	98.4	0.024
**HCV positive household contact (yes vs no)**	3.0	0.4	23.2	0.285	2.8	0.4	21.6	0.315								
**Alcohol addiction (yes vs no)**	2.6	0.3	19.5	0.367	2.4	0.3	18.1	0.402								

*with respect to not infected

**combined estimates of multinomial regression on 10 imputed datasets. Factors not possible to estimate in women: having a tattoo, ever using drugs (intranasal or injecting), HCV positive partner, HCV positive household contact, alcohol addiction

Having a tattoo (AOR 4.6, 95% CI 1.6–13.9), history of injecting drugs (AOR 8.6, 95% CI 1.0–74.0) and history of having an HCV-infected sexual partner (AOR 9.6 95% CI 1.2–79.3) were significantly associated with ‘no significant fibrosis’ in men in multivariable analysis. On the other hand, the history of transfusion prior to 1992 (AOR 12.1, 95% CI 3.7–39.9), and ever having an HCV-infected sexual partner (AOR 11.6, 95% CI 1.4–98.4) predicted significant fibrosis, while history of ever injecting drugs was borderline significant (AOR 8.2, 95% CI 0.9–73.8) (Table 3).

## Discussion

We confirmed a substantial HCV prevalence in Poland with a moderate fraction of HCV infection-related fibrosis assessed by non-invasive methods. Factors which predicted HCV infection with significant liver fibrosis differed from those associated with infection with low fibrosis, although the number of individuals with significant fibrosis was small, limiting detailed analysis. Furthermore, these factors differed between men and women. These findings may be of assistance while designing testing strategies and evaluating the short and long-term effects of these strategies on reducing the burden of HCV infection.

We found that HCV prevalence is similar across all age groups in both adult men and women. This is in contrast to e.g. USA and France, where it is accumulated in specific birth cohorts [10,21]. Consequently, birth cohort screening strategy, introduced in these countries, may not be effective in Poland. Other strategies, including targeted testing, may prove to be more appropriate in terms of case detection and cost-effectiveness.

We found a moderate proportion of cases with significant liver fibrosis. The indication of significant fibrosis was present in 51.6% and 34.4% of HCV-infected men and women, respectively. Only a few studies report the prevalence of hepatitis C by liver fibrosis stage or its distribution in a population based study. Klevens and colleagues reported that over 20% of infections could be associated with significant fibrosis in the USA [22]. Another study confirmed that the average prevalence of intermediate-to-high risk of fibrosis (FIB4 ≥1.45) was 51%, but it increased with time [23]. Bailey and colleagues, who evaluated liver fibrosis in HIV/HCV co-infected pregnant women, found no significant/advanced fibrosis in 47.4% participants by APRI and 74.9% by FIB4 [24]. There are no prior data which could be used to analyse trends in this respect in Poland. Our findings may, however, indicate substantial burden of HCV-related fibrosis.

Significant fibrosis, including compensated cirrhosis, is associated with an increased risk of progression to hepatocellular carcinoma or decompensation. In the study by Xu and colleagues, the proportion of disease progression within 5 years reached 19.6% for F3 and 37.2% for F4 stages compared to 2.3% in case of F0-1 stages [25]. These unfavourable short-term outcomes could be prevented if cases with significant fibrosis were efficiently detected and treated, leading to immediate reduction in the rates of decompensated cirrhosis or cancer [26]. What follows, if the screening programme is designed in a way to maximise the yield of cases with advanced fibrosis, the savings resulting from the prevention of these outcomes could be achieved in a relatively short time. Prompt savings could be in turn allocated for improved prevention, screening and treatment of hepatitis C cases.

The risk factors associated with ‘no-significant’ fibrosis differed from those predicting infections with significant fibrosis. These differences may reflect the natural history of the epidemic, in particular the factors predicting significant fibrosis may be associated with historically important sources of HCV transmission whereas those associated with less fibrosis with more current transmission patterns.

The significant fibrosis was associated with blood transfusion prior to 1992, especially in men (AOR 12.1, 95% CI 3.7–39.9). Routine screening for HCV (anti-HCV) was implemented in 1992. Thus, transfusion prior to 1992 is an established risk factor of chronic hepatitis C in Poland. Interestingly, although transfusion prior to 1992 was also associated with increased odds of significant fibrosis in univariable analysis in women (OR 4.9, 95% CI 3.0–18.5), less such effect was noted in the multivariable model. Blood transfusion prior to 1992 correlated with the number of caesarean sections, which possibly reflects frequent blood loss requiring transfusion, including occasionally massive transfusions (≥8 units of RBC) following caesarean sections [27,28]. Testing of women with a history of multiple caesarean sections, especially prior to 1992, should be considered.

In our study, injecting drug use was associated with increased odds of both no-significant fibrosis (AOR in men 8.6, 95% CI 1.0–74.0) and significant fibrosis (AOR in men 8.2, 95% CI 0.9–73.8) infections. High level of HIV co-infections (29%) among HCV-infected PWID was previously observed [29]. HIV infection contributes to a faster fibrosis progression in HCV-infected patients [30], re-iterating the need for efficient HCV screening in this group. The association between HCV infection and the history of injecting drug use in women could not be estimated due to the fact that the sample of women with a history of injecting drug use was not large enough (29 cases, 1 infected).

Furthermore, we observed the association between HCV infection and the history of sexual contacts with an HCV- infected person (AOR 9.88, 95%CI 2.3–42.51 in women and 7.26, 95%CI 1.59–33.25 in men). Although sexual transmission may not be as effective as other routes, sexual contact with an infected partner and multiple partnerships have been repeatedly found to correlate with higher HCV prevalence [31]. Interestingly, the history of having an HCV-infected partner was associated with both ‘significant fibrosis’ (AOR 11.6, 95% CI 1.4–98.4) and ‘no significant fibrosis’ infections (AOR 9.6, 95% CI 1.2–79.3) in men, possibly suggesting ongoing transmission. Sexual contact is reported to be an efficient HCV transmission route in men who have sex with men (MSM), in particular those infected with HIV [32]. The possibility of ongoing HCV transmission among HIV-infected MSM should be investigated. Due to the small sample of females who reported having an HCV-infected partner (59, 2 infected), the analysis of this factor by fibrosis stage was not possible in this group.

We found that tattooing predicted HCV infection at less advanced stages in men (AOR 4.6, 95% CI 1.6–13.9). Of note, no association was found with more advanced disease, suggesting that this may be an ongoing transmission route or possibly an indicator of other behaviours currently still leading to transmission. In other countries tattooing was found as a risk factor of HCV infection [33,34] although some studies reported no increased risk [33]. The risk was mainly associated with having the tattoo made in non-professional settings including prisons and appeared to decrease since 2000. The associations between tattooing and injecting or snorting drug use were also underlined [33]. In our study, tattooing was present in 10.2% and 3.2% of those aged <44 and 45+, respectively. Recent emergence of this factor may explain why it is associated only with ‘no fibrosis’ infections as these infections are likely of shorter duration. Closer revision of the tattoo procedures should be implemented in order to eliminate the risk of transmission of blood-borne infections through this procedure.

There are three main limitations in our study. Firstly, we relied on the approximate indicators of the stage of HCV infections and our estimate of the stage distribution could be biased. However, these indicators have been validated in many studies and their precision although suboptimal at the clinical level tend to be sufficient for the purpose of epidemiological studies offering a cheaper and logistically feasible alternative to other methods (9,12). We also note that approximately 16% of the country population were not registered in the primary health care and were therefore excluded from the study population. This may be particularly true for marginalized populations such as problem PWID and undocumented migrants, in whom the HCV prevalence is likely to be higher. Thus, the results are generalizable to the general population settings and should be supplemented with targeted monitoring of the high risk populations.

Finally, even though we did have sufficient power to estimate HCV infection prevalence by demographic profile, it has to be acknowledged that our ability to determine the risk factors for “significant fibrosis” and “non-significant fibrosis” infections was already restricted, as evidenced by broad confidence intervals. We can report with some confidence the associations with relatively common HCV risk factors (e.g. transfusion prior to 1992, caesarean section, tattooing), however, the sample size was not large enough to study rare risk factors such as injecting drug use (reported by 0.3% of participants) and ever having an HCV-infected sexual partner (reported by 0.4% of participants). Since these exposures were rare in our study, we conclude they would not strongly affect the possible screening strategy in the general population.

## Conclusions

We showed that HCV infection is prevalent in adults across birth cohorts in Poland. Consequently, it would be not feasible to implement birth cohort based screening. However, if the targeted screening followed by the treatment of HCV cases detected were effectively implemented, especially based on the factors associated with significant fibrosis, we could expect a substantial decrease in the morbidity and mortality rates within several years. Prior research demonstrated effectiveness of targeted screening to identify individuals at a higher risk of HCV infection in the general population [35,36]. Simple recommendations are more likely to be effectuated at the primary care level [37], such as birth cohort screening, but also screening targeted to a single group such as people with the history of injecting drug use [38]. Therefore, we recommend the adoption of fewer, but evidence based, indications for HCV testing in the primary care units. A revision of such strategy after several years is warranted.

## Supporting information

S1 File The original questionnaire (Polish). (PDF)

S2 File The translated questionnaire (English). (PDF)
