# Supplementary material for: Factors associated with hepatitis C prevalence differ by the stage of liver fibrosis: A cross-sectional study in the general population in Poland, 2012-2016
Source: PLoS One. 2017 Sep 20;12(9):e0185055. doi: 10.1371/journal.pone.0185055 (PMC5607182; doi:10.1371/journal.pone.0185055)
Supplement: S1 File — (PDF) [file pone.0185055.s001.pdf]

SWISS CONTRIBUTION

HCV JESTEM ŚWIADOM

Szanowni Państwo!

Zapraszamy do udziału w Projekcie dotyczącym usprawnienia diagnostyki HCV w Podstawowej Opiece Zdrowotnej. Niniejsza ankieta służy zebraniu informacji dotyczących Państwa doświadczeń, które zostaną zanalizowane wraz z wynikami badań laboratoryjnych. Pozwoli to na opracowanie rekomendacji badań przesiewowych w kierunku HCV oferowanych przez lekarzy rodzinnych.

Ankieta jest całkowicie anonimowa. Zebrane informacje nie będą ujawniane i zostaną wykorzystane jedynie w celach naukowych.

Prosimy o udzielenie prawdziwych odpowiedzi. Tylko w ten sposób możemy ustalić ryzyko zakażenia WZW C.

Uwaga: W razie pojawienia się wątpliwości dotyczących pytań zawartych w ankiecie, prosimy o wstrzymanie się z odpowiedzią i skonsultowanie ich z lekarzem prowadzącym.

Ogólny stan zdrowia

1. Czy choruje Pan/i lub kiedykolwiek chorował/a na :

hemofilię	☐ Tak ☐ Nie
cukrzycę	☐ Tak ☐ Nie
niewydolność serca/ chorobę wieńcową	☐ Tak ☐ Nie
astmę/POChP	☐ Tak ☐ Nie
choroby nowotworowe	☐ Tak ☐ Nie
choroby nerek	☐ Tak ☐ Nie
powikłane urazy	☐ Tak ☐ Nie

inną chorobę przewlekłą, z powodu której był/a Pan/i hospitalizowany/a co najmniej 2 razy:

☐ Tak, jaką? ……..…………………… ☐ Nie

2. Czy był/a Pan/i poprzednio badany/a w kierunku WZW C (HCV)?

☐ Tak
☐ Nie
☐ Nie wiem/nie pamiętam

2a. Jeśli TAK, to w jakich okolicznościach?

☐ podczas hospitalizacji (np. przed operacją)
☐ dawca krwi/kandydat na dawcę (po 1992 r.)
☐ nieprawidłowe próby wątrobowe, choroby wątroby, bóle brzucha
☐ stwierdzenie zakażenia u partnera/osoby z otoczenia
☐ badania osób szczególnie narażonych (np. osoby dializowane, osoby uzależnione, po ekspozycji zawodowej)
☐ z własnej inicjatywy
☐ inne okoliczności, jakie?…………………………

3. Czy kiedykolwiek stwierdzono u Pana/i dodatni wynik badania w kierunku HCV?

☐ Tak
☐ Nie
☐ Nie wiem/nie pamiętam

4. Czy chorował/a Pan/i lub był/a zakażony/a wcześniej?

WZW A (żółtaczka pokarmowa)	☐Tak	Rok…….	☐ Nie	☐ Nie wiem
WZW B	☐Tak	Rok…….	☐ Nie	☐ Nie wiem
WZW C	☐Tak	Rok…….	☐ Nie	☐ Nie wiem
HIV	☐Tak	Rok…….	☐ Nie	☐ Nie wiem

Narażenia medyczne

5. Czy kiedykolwiek otrzymywał/a Pan/i transfuzje krwi lub preparatów krwiopochodnych (płytki, osocze, czynniki krzepnięcia, albuminy, immunoglobuliny)?

☐ Nie
☐ Tak, przed 1992 r. ile razy …… jaki preparat ………………..
☐ Tak, po 1992 r. ile razy …… jaki preparat ………………..
☐ Tak, nie pamiętam kiedy ile razy …… jaki preparat ………………..

6. Ile razy przebywał/a Pan/i w szpitalu (w ciągu całego życia pomijając moment narodzin)?
(przyjęcie do szpitala, w tym pobyty jednodniowe i diagnostyczne**)**
☐ nigdy ☐ 1 -2 razy ☐ 3-4 razy ☐ 5 i więcej

7. Czy kiedykolwiek wykonywano u Pana/Pani następujące zabiegi:

zabiegi stomatologiczne	☐ Tak	☐ Nie
endoskopie (np. gastroskopia, kolonoskopia, bronchoskopia)	☐ Tak	☐ Nie
proste zabiegi chirurgiczne (np. szycie rany, nacięcie ropnia, usunięcie znamienia)	☐ Tak	☐ Nie
biopsje	☐ Tak	☐ Nie
cięcie cesarskie	☐ Tak, ile razy …	☐ Nie
zabiegi okołoporodowe (np. cięcie krocza)	☐ Tak, ile razy…	☐ Nie
operacje chirurgiczne (także laryngologiczne, kardiologiczne, ortopedyczne, ginekologiczne inne niż cięcie cesarskie,)	☐ Tak, ile razy…	☐ Nie
inne operacje/ zabiegi medyczne, jakie? …………………	☐ Tak	☐ Nie
dializy	☐ Tak	☐ Nie

8. Czy <u>w ciągu ostatniego roku</u> były wykonywane u Pana/i zastrzyki (nie dotyczy pobrań krwi)?
☐ Tak ☐ Nie

8a. Jeżeli TAK, proszę podać całkowitą liczbę zastrzyków <u>w ciągu ostatniego roku</u> w każdej kategorii:

	**w szpitalu**	**w przychodni**	**w domu**	**w innym miejscu**
leki w zastrzykach (np. antybiotyki, przeciwbólowe, sterydy, witaminy)				
szczepienia				
znieczulenia (także u dentysty)				
kontrast, testy diagnostyczne				
wlewy dożylne (kroplówka)				

Narodowy Instytut Zdrowia Publicznego - Państwowy Zakład Higieny
Instytut Psychiatrii I Neurologii w Warszawie
Uniwersytet Medyczny w Lublinie
Główny Inspektorat Sanitarny

Narażenia pozamedyczne

9. Czy kiedykolwiek korzystał/a Pan/i z niżej wymienionych usług/zabiegów?

tatuaże ☐ Tak, w studiu ☐ Tak, w innych warunkach ☐ Nie
kolczykowanie ☐ Tak ☐ Nie
akupunktura ☐ Tak ☐ Nie
botox/mezoterapia ☐ Tak ☐ Nie
manicure/pedicure w gabinecie kosmetycznym ☐ Tak ☐ Nie
inne zabiegi kosmetyczne lub zabiegi medycyny niekonwencjonalnej, ☐ Tak ☐ Nie
jakie?………………………………………………………………….....

10. Czy był/a Pan/i leczony/a z powodu uzależnienia od alkoholu?
(w oddziale odwykowym/detoksykacyjnym/ grupy AA) ? ☐ Tak ☐ Nie

11. Czy kiedykolwiek przyjmował/a Pan/i narkotyki dożylnie lub donosowo?
☐ Tak, tylko dożylnie ☐ Tak, tylko donosowo ☐ Tak, i donosowo i dożylnie
☐ Nie

12. Czy przebywał/a Pan/i w instytucjach zamkniętych (wychowawczych, w areszcie lub więzieniu) przez czas <u>dłuższy niż 3 miesiące</u>? ☐ Tak ☐ Nie

13. Czy zgodnie z Pana/i wiedzą ktoś z Pana/i domowników obecnie lub w przeszłości:

a.) był/jest zakażony WZW C (HCV)?
☐ Tak stopień pokrewieństwa/znajomości …………….
☐ Nie

b.) Przyjmował/przyjmuje narkotyki dożylnie?
☐ Tak stopień pokrewieństwa/znajomości……………..
☐ Nie

c.) przyjmował/przyjmuje narkotyki donosowo?
☐ Tak stopień pokrewieństwa/znajomości……………..
☐ Nie

14. Czy zgodnie z Pana/i wiedzą którakolwiek z osób, z którymi utrzymuje/utrzymywał Pan/i kontakty seksualne:

jest zakażony WZW C (HCV) ☐ Tak ☐ Nie
przyjmował lub przyjmuje narkotyki dożylnie ☐ Tak ☐ Nie
przyjmował lub przyjmuje narkotyki donosowo ☐ Tak ☐ Nie

Informacje ogólne

15. Płeć: ☐ Kobieta ☐ Mężczyzna

16. Rok urodzenia: |_||_||_||_|

17. Wykształcenie:
☐ Podstawowe/gimnazjum
☐ Zawodowe
☐ Średnie
☐ Policealne
☐ Wyższe/niepełne wyższe

18. Miejsce zamieszkania:
☐ miasto powyżej 100 tys. mieszkańców
☐ miasto 50-99 tys. mieszkańców
☐ miasto 20-49 tys. mieszkańców
☐ miasto < 20 tys. mieszkańców
☐ wieś

19. Liczba osób w gospodarstwie domowym: …………………

20. Miesięczny dochód na członka gospodarstwa domowego netto (średnio w ciągu ostatnich 12 miesięcy):
☐ poniżej 500 zł
☐ 500 – 1000 zł
☐ 1001 – 2500 zł
☐ 2501 – 4000 zł
☐ powyżej 4000 zł

21. Czy obecnie lub wcześniej wykonywał/a Pan/i któryś z poniższych zawodów/prac:

zawód medyczny (z bezpośrednim kontaktem z chorym)	☐ Tak Jaki? ……………… ☐ Nie
sprzątanie/usuwanie odpadów medycznych	☐ Tak ☐ Nie
praca w laboratorium diagnostycznym	☐ Tak ☐ Nie
strażak	☐ Tak ☐ Nie
pracownik służby więziennej	☐ Tak ☐ Nie
pracownik służby porządkowo-prewencyjnej	☐ Tak ☐ Nie
praca z osobami uzależnionymi/bezdomnymi	☐ Tak ☐ Nie

Bardzo dziękujemy za poświęcony czas.

Projekt „Usprawnienie diagnostyki HCV, oszacowanie występowania HCV w populacji ogólnej oraz analiza czynników związanych z występowaniem HCV" w ramach projektu „ Zapobieganie zakażeniom HCV".

Narodowy Instytut Zdrowia Publicznego - Państwowy Zakład Higieny
Instytut Psychiatrii I Neurologii w Warszawie
Uniwersytet Medyczny w Lublinie
Główny Inspektorat Sanitarny
