# Supplementary material for: Factors associated with hepatitis C prevalence differ by the stage of liver fibrosis: A cross-sectional study in the general population in Poland, 2012-2016
Source: PLoS One. 2017 Sep 20;12(9):e0185055. doi: 10.1371/journal.pone.0185055 (PMC5607182; doi:10.1371/journal.pone.0185055)
Supplement: S2 File — (PDF) [file pone.0185055.s002.pdf]

SWISS CONTRIBUTION

HCV JESTEM ŚWIADOM

Dear Sir/Madame,

We would like to invite you to participate in the Project concerning improvement of HCV diagnosis in the primary health care. This questionnaire aims at collecting your experience, which will be analysed together with the test results in order to develop recommendations for provider-initiated testing offered by the primary health care doctors. The questionnaire is fully anonymous. Any identifying information collected will not be released and all the data will be used only for scientific purpose.
We ask you to provide accurate answers. This is the only way to assess the risks of hepatitis C.
Important: In case you have difficulties understanding some of the questions in the questionnaire please consult your doctor.

General health

1. Have you ever suffered from any of the diseases below:

haemophilia	☐ Yes ☐ No
diabetes	☐ Yes ☐ No
heart insufficiency / coronary heart disease	☐ Yes ☐ No
astma/ COPD	☐ Yes ☐ No
cancer	☐ Yes ☐ No
kidney diseases	☐ Yes ☐ No
complicated injuries	☐ Yes ☐ No

other chronic disease, due to which you were hospitalised 2 or more times:
☐ Yes, please list: …………….……..…………………… ☐ No

2. Have you ever been tested for hepatitis C (HCV)?
☐ Yes
☐ No
☐ I don't know / I don't remember

2a. If YES, what were the circumstances?
☐ during hospitalisation (e.g. before surgical procedure)
☐ donating blood (after 1992)
☐ abnormal liver function tests, liver disease, abdominal pain
☐ partner/close contact diagnosed with the infection
☐ screening of risk group (e.g. people on dialysis, drug users, occupational exposure)
☐ own initiative (self-paid)
☐ other circumstances, please list: …………………………………………

3. Have you ever received a positive test result of an HCV test?
☐ Yes
☐ No
☐ I don't know / I don't remember

4. Have you ever been infected or ill with the following diseases?

Hepatitis A	☐Yes	Year …….	☐ No	☐ I don't know
Hepatitis B	☐Yes	Year …….	☐ No	☐ I don't know
Hepatitis C	☐Yes	Year …….	☐ No	☐ I don't know
HIV	☐Yes	Year …….	☐ No	☐ I don't know

Medical exposures

5. Have you ever received blood transfusion (including platelets, plasma, clotting factors, albumines, immunoglobulines)?

☐ No
☐ Yes, before 1992 how many times: …… which blood element: ………………..
☐ Yes, after 1992 how many times: …… which blood element: ………………..
☐ Yes, not remember date how many times: …… which blood element: ………………..

6. How many times have you been in the hospital (lifetime, excluding at birth)?

(please include all admissions to hospital, including one-day stays and diagnostic stays)

☐ never ☐ 1 -2 times ☐ 3-4 times ☐ 5 times and more

7. Have you ever had the following procedures performed on you:

Dental surgery	☐ Yes ☐ No
Endoscopy (e.g. gastroscopy, colonoscopy, bronchoscopy)	☐ Yes ☐ No
Small surgical procedures (e.g. wound sutures, abscess incision, nevus removal)	☐ Yes ☐ No
Biopsy	☐ Yes ☐ No
Caesarean section	☐ Yes:how many times:… ☐ No
Other procedures at delivery (e.g. episiotomy)	☐ Yes:how many times:… ☐ No
Major surgery (also otolaryngologic, cardiologic, orthopedic, gynecologic other that CC)	☐ Yes:how many times:… ☐ No
Other medical procedures, please list: …..………………	☐ Yes ☐ No
Dialysis	☐ Yes ☐ No

8. During the <u>last year</u> did you have any injections (do not include blood collection)?

☐ Yes ☐ No

8a. If YES please estimate the total number of injections <u>during the last year</u> in each category:

	**hospital**	**In patient clinic**	**At home**	**Other places**
Medication by injection (e.g. antibiotics, analgesics, steroids, vitamins)				
Vaccination				
Anesthesia (also at dentist)				
Contrast, diagnostic tests				
i.v. line				

Narodowy Instytut Zdrowia Publicznego - Państwowy Zakład Higieny
Instytut Psychiatrii I Neurologii w Warszawie
Uniwersytet Medyczny w Lublinie
Główny Inspektorat Sanitarny

Non-medical exposures

9. Have you ever had any of the following body modifications /non-medical procedures performed?

tattoo ☐ Yes, professional studio ☐ Yes, other places ☐ No
piercing ☐ Yes ☐ No
acupuncture ☐ Yes ☐ No
botox/mesotherapy ☐ Yes ☐ No
manicure/pedicure in beauty parlour ☐ Yes ☐ No
other cosmetic or non-conventional medicine procedures, ☐ Yes ☐ No
please list:…………………………………………………………………......

10. Have you ever been treated for alcohol addiction?
(in detox/ rehabilitation ward, AA groups) ? ☐ Yes ☐ No

11. Have you ever used illegal drugs by injection or snorting?
☐ Yes, by injection ☐ Yes, snorting ☐ Yes, snorting and injection
☐ No

12. Have you ever been detained for more than <u>3 months</u> (prison, correctional facility, detention centre)?
☐ Yes ☐ No

13. According to your best knowledge, is one of your current of past <u>householders</u>:

a.) Infected with HCV?
☐ Yes – what is your relationship with this person: …………….
☐ No

b.) Injecting drug user?
☐ Yes – what is your relationship with this person: …………….
☐ No

c.) Snorting drug user?
☐ Yes – what is your relationship with this person: …………….
☐ No

14. According to your best knowledge, is one of your current of past sexual partners:

a.) Infected with HCV ☐ Yes ☐ No
b.) Injecting drugs user ☐ Yes ☐ No
c.) Snorting drug user ☐ Yes ☐ No

General information

15. Sex: ☐ Female ☐ Male

16. Year of birth: |_||_||_||_|

17. Education:
☐ Elementary
☐ Occupational
☐ High school
☐ Post high-school
☐ Higher

18. Residence:
☐ city >=100,000 inhabitants
☐ city 50,000-99,999
☐ city 20,000 – 49,999
☐ city <20,000
☐ rural

19. Number of people living in your household: …………………

20. Average monthly net income per household member during the past 12 months:
☐ <500 PLN
☐ 500 – 1000 PLN
☐ 1001 – 2500 PLN
☐ 2501 – 4000 PLN
☐ > 4000 PLN

21. Have you ever worked in one of the following professions?:

Medical profession (direct contact with the patient)	☐ Yes, what profession: ……...………… ☐ No
Cleaning/ removal of medical waste	☐ Yes ☐ No
Work at diagnostic laboratory	☐ Yes ☐ No
Fireman	☐ Yes ☐ No
Prison officer	☐ Yes ☐ No
Police, civil guard etc.	☐ Yes ☐ No
Work with people addicted to drugs or homeless	☐ Yes ☐ No

We would like to thank you for your time.
